# Integrating Individual Placement Support in Early Psychosis Programs: A Necessary Step to Foster Return to Employment in First Episode Psychosis Patients

**DOI:** 10.1111/eip.70195

**Published:** 2026-06-24

**Authors:** M. Renda, P. Golay, D. Spagnoli, C. Bonsack, L. Abrahamyan‐Empson, N. Mebdouhi, L. Alerci, P. Conus

**Affiliations:** ^1^ TIPP Program, Service of General Psychiatry, Department of Psychiatry Lausanne University Hospital Lausanne Switzerland; ^2^ La Source School of Nursing, HES‐SO University of Applied Sciences and Arts Western Switzerland Lausanne Switzerland; ^3^ Faculty of Social and Political Science, Institute of Psychology, University of Lausanne Lausanne Switzerland; ^4^ Service of Community Psychiatry, Department of Psychiatry Lausanne University Hospital Lausanne Switzerland

**Keywords:** first episode psychosis, functional outcome, recovery, supported employment

## Abstract

**Introduction:**

Return to studies or employment, a major element of recovery after a first episode of psychosis (FEP), is out of reach of a significant proportion of patients. Specific programs such as Individual Placement Support (IPS) have been proposed to overcome this hurdle. While some early intervention programs include IPS, others must rely on IPS programs that are open to a wider range of patients. The aim of this paper was to explore the accessibility of IPS for FEP patients treated in such a context.

**Methods:**

One hundred and eighty nine patients of an ongoing FEP cohort study who were treated at our program in the period where the IPS program was available and for whom sufficient data was available were included in the study. We assessed reasons for non‐referral to RESSORT and compared baseline and outcome characteristics of patients who did or did not enter the program.

**Results:**

A small minority of patients had access to RESSORT. Patients who entered RESSORT had better premorbid functioning and a higher degree of insight at baseline than the ones who did not enter the program. Patients who entered RESSORT had a significantly higher rate of return to premorbid PAS score, without significant difference on other dimensions of functioning.

**Conclusions:**

When IPS programs are not embedded in early intervention programs, FEP patients have limited chances to access them. Integrating supported employment skills within the role of case managers may be a solution in programs where resources are lacking to develop an IPS program specifically dedicated to FEP patients.

## 1 Introduction

A large body of research shows that functional outcome and capacity to work remains poor among patients with psychosis (Office fédéral de la statistique. Confédération Suisse 2024; Office Fédéral de la Statistique 2024; Carmona et al. 2017). This might be linked to the fact that in 85% of cases, the first symptoms appear between the ages of 15 and 30 (Schizinfo 2024; Humensky et al. 2019; Hygiene GM and Psychose: De l'importance d'une détection précoce—Planete sante 2023), a critical period for the acquisition of numerous competences and the definition of one's professional trajectory. Without optimal care, a psychotic disorder emerging during this period of life wears the risk to lead to a chronic illness with negative consequences especially at the functional level (Humensky et al. 2019; Aguey‐Zinsou et al. 2023; Dutoit et al. 2014; Abdel‐Baki et al. 2013; Baksheev et al. 2012; Williams et al. 2023; Jäckel et al. 2023). This translates into very low rates of employment among persons with psychosis (Humensky et al. 2019; Aguey‐Zinsou et al. 2023; Dutoit et al. 2014; Abdel‐Baki et al. 2013; Baksheev et al. 2012; Williams et al. 2023; Jäckel et al. 2023; Jones et al. 2023; Sveinsdottir et al. 2020), which is really unfortunate since work has been shown to be an asset for recovery (e.g., by enabling financial independence, improving self‐esteem, promoting cognitive and mental health improvements, and fostering social contacts (Aguey‐Zinsou et al. 2023; Dutoit et al. 2014; Baksheev et al. 2012; Burns and Erickson 2023; Frederick and VanderWeele 2019; Carmona et al. 2019; McGahey et al. 2016; Von Peter et al. 2021; Butenko et al. 2023; Williams et al. 2015)) and is included by many patients as one of their primary life goals (Aguey‐Zinsou et al. 2023; Dutoit et al. 2014; Jäckel et al. 2023; Jones et al. 2023; Burns and Erickson 2023; Butenko et al. 2023; Williams et al. 2015; Brinchmann et al. 2020). Although early intervention strategies proposed by specialised programs may have a positive impact in this regard (Santesteban‐Echarri et al. 2017), specific support is needed to improve access or return to work or studies.

The development of Individual Placement and Support programs (IPS) in the 90's has been proposed as a potential solution to this problem (Williams et al. 2015; Drake and Bond 2023). Unlike previous approaches which were based on the principle to first train then place, the IPS approach proposes conversely to first place and then train (Frederick and VanderWeele 2019). The program is based on eight fundamental principles: (1) Rapid job search; (2) Respect for the patient's employment preferences; (3) Competitive employment goal; (4) Zero exclusion policy; (5) Long‐term individual support; (6) Partnership between employment services and mental health services; (7) Personalised advice on social benefits and (8) Creation of a network of potential employers (Drake and Bond 2023). Currently, among all employment support programs, IPS has the best results in enabling beneficiaries to work more hours, more days and earn higher wages than those who do not benefit from it (Baksheev et al. 2012; Frederick and VanderWeele 2019; Simmons et al. 2023). This program also has the best cost–benefit ratio for society (Drake and Bond 2023).

In 2009, an IPS program was created in Lausanne, Switzerland. The RESSORT program (RESeau de Soutien et d'Orientation vers le Travail or Support and Guidance Network for Work) (Dutoit et al. 2014) was launched in the frame of the fifth revision of the disability insurance system, which aimed to reduce the number of new disability benefits and to promote professional reintegration (Dutoit et al. 2014; Spagnoli 2018). As shown in the literature, IPS implemented in the early stages of psychiatric illness has many advantages, notably to reduce the period of unemployment and its negative social impact (Abdel‐Baki et al. 2013; Baksheev et al. 2012; Bond et al. 2015). When RESSORT was launched, the hope was therefore to make it accessible to patients who need supported employment enrolled in TIPP (Treatment and Intervention in the Early Phase of Psychotic Disorders), a 3 year first episode psychosis program that was already in place in Lausanne since 2004 (Bond et al. 2015; Conus et al. 2010; CHUV Département de Psychiatrie 2024), and this although it was not restricted solely to patients treated in this program.

RESSORT received financial support from the Disability Insurance Office and the Social Welfare and Assistance Service (Dutoit et al. 2014; Spagnoli 2018) due to their converging aims. RESSORT currently consists both of an employment support program for people with severe mental illnesses and an engagement to care program for people with difficulties at work suggesting a mental disorder (Dutoit et al. 2014; Spagnoli 2018; CHUV Département de Psychiatrie 2023). The former offers assistance to patients who wish to return to work or continue their education, regardless of the severity of their underlying condition, while the latter works with social services to refer beneficiaries to the appropriate institution when a psychiatric condition is suspected to be hindering their reintegration (Dutoit et al. 2014; Spagnoli 2018; CHUV Département de Psychiatrie 2023).

Considering these elements and the observation by Drake et al. (Drake et al. 2016) that IPS is still unavailable to a large majority of people with serious mental illnesses in the United States, we wanted to explore the accessibility and suitability of our IPS setting for patients treated at TIPP. Our hypothesis was that since RESSORT was open to a large range of patients, its accessibility to TIPP patients was likely to be very limited. More precisely, we wanted to study first the access to RESSORT for TIPP patients, second the characteristics of TIPP patients who were not referred to RESSORT by their case manager and the reason why they were not referred to this program, third the profile of patients who, despite being referred to RESSORT, did not enter the program. Finally, among TIPP patients who were sent to RESSORT, we wanted to compare the outcome between those who entered this program and those who did not.

## 2 Methods

This paper is based on a quasi‐experimental prospective cohort design comparing patients who were referred to the RESSORT program with those who weren't. The assessment of referral is based on an audit of both TIPP and RESSORT patients' lists.

All patients enrolled in TIPP are assessed upon entry into the program (baseline assessment) and then prospectively every 6 months, up to 36 months, to identify risk factors for adverse outcomes and characteristics that may guide treatment and to evaluate response to treatment. During the baseline assessment, a specific questionnaire is completed by case managers based on information provided by patients and their relatives. This baseline questionnaire can be completed and modified throughout the follow‐up period, whenever additional data is emerging, which is not uncommon considering the need for a trusting relationship to talk about issues such as exposure to trauma or substance use, for example.

At each follow‐up assessment, at months 6, 12, 18, 24, 30 and 36, data is provided both by case managers for factual scales (such as employment, living situation for example) and by a clinical psychologist who is conducting psychometric scale‐based assessments (see below). These data are transferred in a database which contains demographic and contextual characteristics as well as the results of psychopathological and psychosocial functioning assessments, and the Vaud Cantonal Research and Ethics Committee has granted us access to these clinical data for research purposes if patients give their consent.

### 2.1 Diagnostic Assessment

Diagnosis, based on an expert consensus carried out by a senior psychiatrist and the senior psychologist who conducts scale‐based assessment over the treatment period, is based on the following elements: (1) the diagnoses found in the medical file and at the end of any hospitalisation and (2) the longitudinal assessment by clinical case managers over the 3 years of treatment. They both review the entire file at two timepoints: after 18 months and after 36 months in treatment. The final diagnosis is defined based on the *Diagnostic and Statistical Manual of Mental Disorders* (DSM‐IV) criteria, after discussion with the case manager if there are any unclear issues.

### 2.2 Pre‐Treatment Characteristics

Premorbid functioning was assessed using the Premorbid Adjustment Scale (PAS) (Cannon‐Spoor et al. 1982), and data were used to compute a global, an academic and a social sub‐score, as well as scores in childhood and in the early adolescence phases (Alameda et al. 2015). Duration of untreated psychosis (DUP), defined as the time between the onset of psychotic symptoms and the date of entry into the TIPP program, was estimated by expert consensus based on information gathered from the patient and relatives (Polari et al. 2011). Past psychiatric diagnoses were determined according to DSM‐IV criteria, and past suicide attempts were based on the ICD‐10 classification (Dilling and Dittmann 1990). Case managers identified a past history of trauma (physical, emotional or sexual abuse) based on their knowledge of the patient's history within a 3‐year therapeutic relationship and interviews with relatives, when indicated (Alameda et al. 2015). Socio‐economic status (SES) was categorised into high, intermediate, and low according to Chandola and Jenkinson (Chandola and Jenkinson 2000). Lifetime diagnosis of substance abuse or dependence before entering the program was rated by case managers based on DSM‐IV (American Psychiatric Association 2000). History of migration in adversity was considered when patients had come to Switzerland as political or economic refugees.

### 2.3 Patient Characteristics at Baseline

Age was recorded in years, gender within two categories (male or female), and marital status was divided into four categories: single, married, divorced or de‐facto. Patients were rated as ‘working’ at baseline using the Modified Vocational Status Index (MVSI) (Tohen et al. 2000), if they fulfilled one of the following characteristics: paid or unpaid full−/part‐time employment, active student status, head of household with an employed partner, or part−/full‐time volunteer work. They were rated as ‘living independently’, based on the Modified Location Code Index (MLCI) (Tohen et al. 2000) if they either were living alone, with a partner or peers, or with family under minimal supervision.

Functioning level at baseline was assessed using both the Global Assessment of Functioning scale (GAF) (American Psychiatric Association 2000) and the Social and Occupational Functioning Assessment Scale (SOFAS) (American Psychiatric Association 2000): while SOFAS focuses specifically on social/occupational functioning, GAF includes symptom intensity. Severity of illness at baseline was measured with the Clinical Global Impression Scale (CGI) (Guy 1976). Considering some patients could have been exposed to medication at the emergency or in hospital in the few days preceding their baseline assessment, a consensus rating of the worse scores on GAF, SOFAS, and CGI was made by clinicians based on all information that was available when seeing the patient for the first time. Awareness of the illness (or insight) was rated by case managers as absent, partial or complete, based on their very close and frequent interaction with patients and discussion about their level of awareness of the presence of their psychotic disorder.

### 2.4 Characteristics Over the Treatment Period and Outcome

Suicide attempts and episodes of legal offences were recorded by case managers. Episodes of hospitalisation during the program were divided into three levels: no hospitalisation, one hospitalisation or multiple hospitalisations. Type of follow‐up after discharge from TIPP was subdivided as follows: specialised outpatient care, other outpatient care, psychiatrist or psychologist in private practice, general practitioner, no follow‐up necessary or other.

Symptomatic remission was defined according to Andreasen's Criteria (Portal de Periódicos da CAPES 2025): ratings of mild or less (≤ 3) on specific PANSS items (delusions, unusual thought content, hallucinations, conceptual disorganisation, mannerisms, blunted affect, social withdrawal, lack of spontaneity) maintained over 6 months.

Functional recovery was assessed in five different ways: First, we defined functional recovery as final PAS scores lower than or equal to the premorbid rating on at least four of the five items of the PAS general scale (as proposed by Strakowski et al. 1998). It included independence development, global functioning, socio‐personal adjustment, degree of interest in life, and energy level. Ratings on education and abruptness in the change in work were not included in this definition, as they could not have changed during the outcome period (Conus et al. 2006). Second, as a GAF score ≥ 60 at the last assessment. Third, based on the MVSI with scores reflecting independent work. Fourth, based on the MLCI, by scores reflecting independent living; and fifth, with combined MLCI and MVSI scores suggesting combined independent work and living. Disengagement was defined as patients who were lost to follow‐up despite assertive attempts to engage them in treatment and regular visits at home.

### 2.5 Access to RESSORT for TIPP Patients

To explore accessibility to IPS for TIPP patients, we explored the following elements. First, we assessed which were the actual access criteria to RESSORT. Second, we assessed which criteria were applied by case managers to select patients they would refer or not to RESSORT. Third, we counted how many patients of the sample were referred to RESSORT by TIPP case managers. Fourth, among TIPP patients who were sent to IPS by their case manager, we assessed how many did not actually enter RESSORT and for which reason. Fifth, we performed a statistical analysis to compare the demographic, symptomatic, and functional characteristics of TIPP patients referred to RESSORT with those who were not referred to that program. Finally, we performed a statistical analysis to compare, among TIPP patients who had been referred to RESSORT, the characteristics of those who did enter the program and those who did not.

### 2.6 Statistical Analysis

To perform statistical analyses, the following non‐parametric tests were used depending on the nature of the dependent variable: the *T*‐test for continuous dependent variables and the Chi‐square test (or Fischer's exact test when appropriate) for categorical dependent variables. For longitudinal variables, the MMRM (*mixed effects models repeated measures analysis of variance*) method was applied. αStatistical significance was set at the 0.05 significance level. The analyses were performed using IBM SPSS version 27 software.

## 3 Results

### 3.1 Sample and Access to RESSORT

Exploration of the inclusion criteria to RESSORT applied at the time of the study revealed some elements that may limit access to several patients in the early phases of psychosis. Indeed, RESSORT was not accessible to students in need of support in education, patients with a work capacity inferior to 50% as estimated by case managers, patients without any concrete wish to return to work, as well as people without work permits such as illegal migrants.

Between 2013 and 2021, 470 patients entered the TIPP program. Considering we wanted to perform a very detailed analysis of potential differences between groups including differences at the level of symptoms, 281 patients who had not gone through PANSS assessments could not be included in the study; we were therefore able to analyse data from 189 patients who met the inclusion criteria for the TIPP program and for whom complete data were available.

Of these 189 patients, 151 were not referred to RESSORT by the TIPP case managers. Among the 38 patients referred to RESSORT, 15 entered the program, 2 were awaiting a response at the time of data analysis and 21 did not enter the RESSORT program. None of the 281 patients excluded from the study due to lack of clinical data entered the RESSORT program.

Considering the low percentage of TIPP patients who were sent to RESSORT, we compared patients who were referred to RESSORT and those who weren't, based on the whole sample. Patients being sent to RESSORT had a higher CGI score at baseline (4.70 (SD = 1.36) versus 3.48 (SD = 1.34), t (25.679) = 4.194, *p* < 0.001). Comparison of the GAF baseline score revealed the same pattern (40.77 (SD = 17.00) versus 54.23 (SD = 17.81), t(412) = −4.687, *p* < 0.001). Therefore, across the full sample without exclusions, patients referred to RESSORT demonstrated higher functioning, consistent with findings observed in the more restricted sample.

### 3.2 Characteristics of Patients Who Were Not Referred to RESSORT by Case Managers and Reasons for Not Referring Them to This Program

Among the 189 patients included in the analysis, 151 were not referred to the RESSORT program by case managers for one or more of the following reasons: 33% were active students, 72% had a disability benefit application pending, 13% were undocumented, 57% did not have an estimated working capacity of at least 50%, and 56% did not have a concrete wish to return to work.

At baseline, patients who were referred to RESSORT had a significantly higher level of functioning compared to those who were not referred to this program, both at the level of GAF (55.56 vs. 48.21, *p* = 0.019) and SOFAS (54.69 vs. 47.90, *p* = 0.022). We did not find any other significant difference between groups at entry to the TIPP program (See Table 1).

**TABLE 1:** Patients from TIPP sent to RESSORT.

Variable	Not sent to supported employment	Sent to supported employment	Statistic	*p*
	*N* = 151 (79.9%)	*N* = 38 (20.1%)		
Age in years, M (SD)	24.71 (4.86)	24.79	t(187) = −0.093	0.926
Gender, Male, % (*N*)	68.9 (104)	76.3 (29)	c^2^ (1) = 0.806	0.369
Duration of untreated psychosis in days, median (IQR)^a^	61.00 (242.00)	65.50 (185.00)	t(187) = 0.353	0.724
Socio‐economic level, % (*N*)				
Low	27.2 (41)	21.1 (8)	c^2^ (2) = 2.681	0.262
Intermediate	33.1 (50)	47.4 (18)		
High	39.7 (60)	31.6 (12)		
Age of onset in years, M (SD)	23.57 (5.13)	23.53 (4.50)	t(187) = 0.047	0.962
Education in years, M (SD)	10.95 (2.49)	11.03 (2.60)	t(143) = −0.167	0.868
Marital status, % (*N*)				
Single	85.7 (126)	94.6 (35)	Fischer exact test	0.733
Married	8.8 (13)	5.4 (2)		
Divorced	2.0 (3)	0.0 (0)		
De‐facto	3.4 (5)	0.0 (0)		
Professional activity, % (*N*)				
Unemployed	42.2 (62)	47.4 (18)	Fischer exact test	0.919
Full‐time job	3.4 (5)	5.3 (2)		
Student/Internship	20.4 (30)	18.4 (7)		
Part‐time job	4.1 (6)	5.3 (2)		
Disability annuity	1.4 (2)	0.0 (0)		
On sick leave	28.6 (42)	23.7 (9)		
Lifestyle, % (*N*)				
Family	46.3 (68)	60.5 (23)	Fischer exact test	0.614
Independent household	15.0 (22)	13.2 (5)		
With friends	27.9 (41)	18.4 (7)		
Pension/care home	6.1 (9)	2.6 (1)		
Unsettled (hotel, homeless shelter)	4.8 (7)	5.3 (1)		
Premorbid Adj. (PAS) M (SD)				
Childhood	0.27 (0.18)	0.26 (0.17)	t(163) = 0.129	0.898
Early adolescence	0.31 (0.17)	0.27 (0.17)	t(160) = 1.263	0.208
Social	0.26 (0.21)	0.21 (0.18)	t(157) = 1.395	0.165
Academic	0.33 (0.20)	0.35 (0.22)	t(164) = −0.402	0.688
Total	0.29 (0.17)	0.27 (0.16)	t(150) = 0.663	0.508
Past suicide attempt, % (*N*)	15.1 (22)	10.5 (4)	c^2^ (1) = 0.513	0.474
Suicide attempt during program, % (*N*)	9.2 (12)	8.8 (3)	Fischer exact test	1
History of trauma^b^, % (*N*)	39.3 (59)	39.5 (15)	c^2^ (1) = 0.000	0.987
Migration in adversity, % (*N*)	18.5 (28)	7.9 (3)	c^2^ (1) = 2.511	0.113
Forensic history, % (*N*)	15.6 (19)	26.7 (8)	c^2^ (1) = 2.028	0.154
Offences during program, % (*N*)	9.1 (12)	8.1 (3)	Fischer exact test	1
Psychiatric history, % (*N*)	50.0 (71)	60.5 (23)	c^2^ (1) = 1.331	0.249
Family psychiatric history, % (*N*)	51.1 (70)	37.1 (13)	c^2^ (1) = 2.173	0.140
Family history of schizophrenia, % (*N*)	15.7 (21)	13.9 (5)	c^2^ (1) = 0.070	0.792
Lifetime substance abuse (DSM), % (*N*)				
Alcohol	10.6 (16)	7.9 (3)	Fisher's exact test	0.769
Cannabis	19.2 (29)	21.1 (8)	c^2^ (1) = 0.066	0.798
Other substances	4.6 (7)	2.6 (1)	Fischer exact test	1
Lifetime substance addiction (DSM), % (*N*)				
Alcohol	2.6 (4)	0.0 (0)	Fischer exact test	0.585
Cannabis	15.9 (24)	18.4 (7)	c^2^ (1) = 0.141	0.707
Other substances	4.0 (6)	2.6 (1)	Fischer exact test	1
Insight at presentation, % (*N*)				
Absent	22.6 (33)	18.9 (7)	c^2^ (2) = 0.252	0.882
Partial	50.0 (73)	51.4 (19)		
Complete	27.4 (40)	29.7 (11)		
GAF, M (SD)				
Program entry	48.21 (16.14)	55.56 (17.74)	t(168) = −2.374	0.019
Worst during psychosis	29.42 (11.37)	31.76 (11.33)	t(181) = −1.115	0.266
SOFAS, M (SD)				
Program entry	47.90 (15.32)	54.69 (16.76)	t(168) = −2.317	0.02
Worst during psychosis	30.89 (11.02)	31.30 (10.85)	t(180) = −0.201	0.841
CGI, M (SD)				
Program entry	3.91 (1.21)	3.30 (1.26)	t(84) = 1.951	0.054
Higher during psychosis	5.53 (0.78)	5.45 (0.61)	t(82) = 0.429	0.669
Diagnosis, % (*N*)				
Schizophrenia	43.7 (66)	52.6 (20)	Fischer exact test	0.816
Schizophreniform/brief	20.5 (31)	15.8 (6)		
Schizoaffective	13.9 (21)	7.9 (3)		
Major depression^c^	5.3 (8)	5.3 (2)		
Bipolar disorder	7.3 (11)	5.3 (2)		
Other	9.3 (14)	13.2 (5)		
Program commitment, % (*N*)				
Lost from sight	7.2 (6)	0.0 (0)	Fischer exact test	0.334
Follow‐up after program, % (*N*)				
Specialised outpatient care	28 (21)	20.0 (5)	Fischer exact test	0.824
Other outpatient care	14.7 (11)	16.0 (4)		
Private practice psychiatrist/psychologist	37.3 (28)	52.0 (13)		
General practitioner	9.3 (7)	8.0 (2)		
No follow‐up needed	5.3 (4)	0.0 (0)		
Other	5.3 (4)	4.0 (1)		
Symptomatic response at the last assessment of the last year of the program (Andreassen), % (*N*)	53.0 (44)	75.0 (15)	c^2^ (1) = 3.184	0.074
Functional recovery (PAS) at the last assessment of the last year of the program, % (*N*)	44.3 (47)	32.1 (9)	c^2^ (1) = 1.354	0.244
Functional recovery (GAF ≥ 60), % (*N*)	56.5 (48)	50.0 (15)	c^2^ (1) = 0.375	0.540
Functional recovery—independent work, % (*N*)	44.0 (37)	43.3 (13)	c^2^ (1) = 0.005	0.946
Functional recovery—independent living, % (*N*)	72.9 (62)	56.7 (17)	c^2^ (1) = 2.731	0.098
Combined functional recovery (independent work & living), % (*N*)	38.1 (32)	30.0 (9)	c^2^ (1) = 0.629	0.428
Hospitalisations during program, % (*N*)				
None	28.5 (37)	20.6 (7)	c^2^ (2) = 1.200	0.549
One	30.0 (39)	38.2 (13)		
Several	41.5 (54)	41.2 (14)		

Abbreviations: IQR, interquartile range; Mdn, median; Ref.cat, reference category.

^a^ Raw data are presented; however, the test statistics were based on log10 (+constant) transformed data because of extreme positive skewness.

^b^ Physical, emotional, or sexual abuse.

^c^ With psychotic features.

### 3.3 Reasons for Not Entering the Program RESSORT

Of the 38 patients referred to RESSORT by TIPP case managers, 21 did not enter the program for the following reasons: *1 was too unstable (as defined by the case manager based on the presence of symptoms hindering* the *patient's capacity to collaborate on a project or limited engagement requiring home visits*, *for example)*, 1 had insurance issues, 1 had a project that did not meet RESSORT's specifications, 2 were finally not interested in RESSORT, 9 had obtained disability benefits or were working on a project in collaboration with disability services and 1 patient had died. For 2 of them, no information was available.

### 3.4 Comparison Between Referred Patients Who Did or Not Enter the Program RESSORT

Among the 38 patients referred to RESSORT, those who entered the program had better scores than those who did not in the following areas: premorbid adjustment in childhood (0.16 vs. 0.32, *p* = 0.005), premorbid academic adjustment (0.25 vs. 0.39, *p* = 0.047), level of insight upon entry into the TIPP program (absent: 21.4% vs. 19.0%, partial: 21.4% vs. 71.4%, complete: 57.1% vs. 9.5%, *p* = 0.004). The outcome level of functional recovery defined as PAS score equal to or lower than premorbid levels on 4 of 5 PAS general items at the last assessment in the final year of the program was also better (60.0% vs. 17.6%, *p* = 0.039) (See Table 2). They did not differ in the four other measures of functional recovery based on GAF, SOFAS, MLCI and MVSI.

**TABLE 2:** Patients from TIPP accepted into RESSORT.

Variable	Not accepted supported employment	Accepted supported employment	Statistic	*p*
	*N* = 21 (58.3%)	*N* = 15 (41.7%)		
Age in years, M (SD)	25.33 (4.92)	24.33 (4.25)	t(34) = 0.635	0.530
Gender, Male, % (*N*)	81.0 (17)	66.7 (10)	Fischer exact test	0.443
Duration of untreated psychosis in days, Mdn (IQR)^a^	65.00 (317.00)	48.00 (181.00)	t(34) = 0.311	0.758
Socio‐economic level, % (*N*)				
Low	19.0 (4)	26.7 (4)	Fischer exact test	0.748
Intermediate	52.4 (11)	40.0 (6)		
High	28.6 (6)	33.3 (5)		
Age of onset in years, M (SD)	23.67 (4.63)	23.47 (4.45)	t(34) = 0.130	0.897
Education in years, M (SD)	10.82 (2.58)	11.46 (2.73)	t(28) = −0.655	0.518
Marital status, % (*N*)				
Single	95.2 (20)	92.9 (13)	Fischer exact test	1.000
Married	4.8 (1)	7.1 (1)		
Divorced	0.0 (0)	0.0 (0)		
De‐facto	0.0 (0)	0.0 (0)		
Professional activity, % (*N*)				
Unemployed	61.9 (13)	33.3 (5)	Fischer exact test	0.541
Full‐time job	4.8 (1)	6.7 (1)		
Student/Internship	9.5 (2)	20.0 (3)		
Part‐time job	4.8 (1)	6.7 (1)		
Disability annuity	0.0 (0)	0.0 (0)		
On sick leave	19.0 (4)	33.3 (5)		
Lifestyle, % (*N*)				
Family	57.1 (12)	66.7 (10)	Fischer exact test	0.590
Independent household	9.5 (2)	20.0 (3)		
With friends	23.8 (5)	6.7 (1)		
Pension/care home	4.8 (1)	0.0 (0)		
Unsettled (hotel, homeless shelter)	4.8 (1)	6.7 (1)		
Premorbid Adj. (PAS) M (SD)				
Childhood	0.32 (0.15)	0.16 (0.15)	t(32) = 3.046	0.005
Early adolescence	0.29 (0.14)	0.23 (0.18)	t(32) = 1.072	0.292
Social	0.23 (0.17)	0.16 (0.18)	t(32) = 1.150	0.259
Academic	0.39 (0.23)	0.25 (0.16)	t(31) = 2.066	0.047
Total	0.31 (0.14)	0.20 (0.16)	t(31) = 2.010	0.053
Past suicide attempt, % (*N*)	4.8 (1)	20.0 (3)	Fischer exact test	0.287
Suicide attempt during program, % (*N*)	5.6 (1)	14.3 (2)	Fischer exact test	0.568
History of trauma^b^, % (*N*)	38.1 (8)	46.7 (7)	c^2^ (1) = 0.264	0.607
Migration in adversity, % (*N*)	4.8 (1)	13.3 (2)	Fischer exact test	0.559
Forensic history, % (*N*)	29.4 (5)	16.7 (2)	Fischer exact test	0.665
Offences during program, % (*N*)	10.0 (2)	6.7 (1)	Fischer exact test	1
Psychiatric history, % (*N*)	71.4 (15)	46.7 (7)	c^2^ (1) = 2.258	0.133
Family psychiatric history, % (*N*)	30.0 (6)	42.9 (6)	Fisher's exact test	0.487
Family history of schizophrenia, % (*N*)	14.3 (3)	14.3 (2)	Fisher's exact test	1
Lifetime substance abuse (DSM), % (*N*)				
Alcohol	9.5 (2)	6.7 (1)	Fisher's exact test	1
Cannabis	23.8 (5)	20.0 (3)	Fischer exact test	1
Other substances	0.0 (0)	6.7 (1)	Fischer exact test	0.417
Lifetime substance addiction (DSM), % (*N*)				
Alcohol	0.0 (0)	0.0 (0)	NA	NA
Cannabis	19.0 (4)	20.0 (3)	Fischer exact test	1.0
Other substances	0.0 (0)	6.7 (1)	Fischer exact test	0.417
Insight at presentation, % (*N*)				
Absent	19.0 (4)	21.4 (3)	Fischer exact test	0.004
Partial	71.4 (15)	21.4 (3)		
Complete	9.5 (2)	57.1 (8)		
GAF, M (SD)				
Program entry	53.45 (13.93)	60.36 (22.3)	t(32) = −1.113	0.274
Worst during psychosis	31.10 (8.61)	32.13 (14.03)	t(33) = −0.269	0.789
SOFAS, M (SD)				
Program entry	52.70 (13.28)	59.71 (20.73)	t(20.4) = −1.116	0.277
Worst during psychosis	30.70 (8.31)	31.80 (13.73)	t(33) = −0.294	0.770
CGI, M (SD)				
Program entry	3.11 (1.27)	3.40 (1.35)	t(17) = −0.479	0.638
Higher during psychosis	5.33 (0.50)	5.60 (0.70)	t(17) = −0.946	0.357
Diagnosis, % (*N*)				
Schizophrenia	57.1 (12)	40.0 (6)	Fischer exact test	0.689
Schizophreniform/brief	14.3 (3)	20.0 (3)		
Schizoaffective	4.8 (1)	13.3 (2)		
Major depression^c^	4.8 (1)	6.7 (1)		
Bipolar disorder	9.5 (2)	0.0 (0)		
Other	9.5 (2)	20.0 (3)		
Program commitment, % (*N*)				
Lost from sight	0.0 (0)	0.0 (0)	NA	NA
Follow‐up after program, % (*N*)				
Specialised outpatient care	9.1 (1)	23.1 (3)	Fischer exact test	0.597
Other ambulatory care	18.2 (2)	15.4 (2)		
Private practice psychiatrist/psychologist	54.5 (6)	53.8 (7)		
General practitioner	18.2 (2)	0.0 (0)		
No follow‐up needed	0.0 (0)	0.0 (0)		
Other	0.0 (0)	7.7 (1)		
Symptomatic response at the last assessment of the last year of the program (Andreassen), % (*N*)	81.8 (9)	75.0 (6)	Fischer exact test	1
Functional recovery (PAS) at the last assessment of the last year of the program, % (*N*)	17.6 (3)	60.0 (6)	Fischer exact test	0.039
Functional recovery (GAF ≥ 60), % (*N*)	50.0 (7)	57.1 (8)	c^2^ (1) = 0.144	0.705
Functional recovery—independent work, % (*N*)	28.6 (4)	50.0 (7)	c^2^ (1) = 1.348	0.246
Functional recovery—independent living, % (*N*)	42.9 (6)	71.4 (10)	c^2^ (1) = 2.333	0.127
Combined functional recovery (independent work & living), % (*N*)	21.4 (3)	35.7 (5)	Fischer exact test	0.678
Hospitalisations during program, % (*N*)				
None	22.2 (4)	21.4 (3)	Fischer exact test	0.208
One	27.8 (5)	57.1 (8)		
Several	50.0 (9)	21.4 (3)		

Abbreviations: IQR, interquartile range; Mdn, median; Ref.cat, reference category.

^a^ Raw data are presented; however, the test statistics were based on log10 (+constant) transformed data because of extreme positive skewness.

^b^ Physical, emotional, or sexual abuse.

^c^ With psychotic features.

## 4 Discussion

The aim of this study was to explore to which degree first episode psychosis patients accessed IPS when it is not directly embedded within the early intervention program and when it is open to a wider range of subjects with psychiatric disorders. In addition, we wanted to identify the characteristics of FEP patients who accessed the program and the impact it had on their outcome. There are three main results stemming from our data analysis.

First, a very small minority of TIPP patients had access to RESSORT. Second, TIPP patients who entered RESSORT had better premorbid functioning and a higher degree of insight at baseline than the ones who did not enter the program. Third, patients who entered RESSORT had a significantly higher rate of return to premorbid PAS score, without a significant difference on other dimensions of functioning. These observations will be discussed below.

As hypothesised, very few people in the early psychosis program accessed RESSORT. Requests to the IPS program represented approximately 10% of patients from the TIPP early psychosis program and only half of them (55%) ultimately accessed the IPS program. As a result, less than 5% of patients in the early psychosis program access standard IPS‐type employment support. Difficulties in accessing the program may be related to the situation of the target population, to the functioning of the early psychosis and IPS programs, or to the socio‐economic and social benefits contexts.

In the initial phase of psychosis, the issue of employment may not be considered a priority in care settings: care focuses on engaging in a recovery process, establishing appropriate treatment, and providing information to patients and families. Many patients may be considered unfit for work during this period. It is likely that many first episode psychosis patients have not finished any training, have gone through very difficult moments and have lost confidence in themselves: in such a context, it is probably very difficult for them to display a great motivation to work and to convince anyone that they could perform if they were to have a job. Moreover, applications to the disability insurance scheme for a pension or for professional reintegration must be submitted quickly, according to Swiss laws, to protect patients' rights to these social benefits. Therefore, the requirement for a minimum 50% activity rate or the absence of a current application to the disability insurance scheme for admission to the employment support program can be dissuasive for this population. These criteria may also have refrained TIPP case managers from referring patients to RESSORT. Furthermore, in the Swiss context, direct access to employment is not valued without certification of professional education or internships: supported education is therefore often more necessary than supported employment. The observation that one third of patients were excluded because they were students shows that IPS programs dealing with first episode psychosis patients should also include supported education in their objectives. However, when comparing patients referred to RESSORT by case managers and those who were not, the only significant differences at baseline were found at the level of functional assessment by GAF and SOFAS, showing that it was not linked to the diagnostic subtype of persistent symptoms or the presence of substance abuse for example. This suggests that the zero‐exclusion principle was applied for the eligible patients.

The10% of patients in the TIPP program who were referred to RESSORT had higher premorbid and current functioning. However, half of them ultimately did not access the program; most were finally diverted to disability insurance benefits, which, through its vocational integration program for young people, offers greater benefits than the IPS program both for individuals and employers and includes supported education. For the others, the obstacles were the lack of an employment project, requirement for 50% activity, or the instability of health status. Only a minority were rejected because of clinical instability of symptoms (5%).

The observation that only a very small minority of TIPP patients (3%) had access to RESSORT is nevertheless concerning, considering the proven impact of such programs in the early phase of psychosis. These numbers suggest that when IPS is not dedicated to psychosis patients, their chances to access this useful resource are very limited.

When comparing patients who did or did not enter RESSORT, we found that the first had better premorbid functioning and a higher degree of insight. Functional outcome at the end of treatment at TIPP was significantly better in one measure (return to premorbid PAS level) but not on the other functional assessments (GAF, SOFAS, MLCI, MVSI). This suggests that the complementary offer by disability insurance may also be effective for some functional dimensions. Indeed, we also observed that some TIPP patients were referred to the disability pension program although they had a low level of symptoms and a higher functional level and were eligible for RESSORT. This suggests that this entity, which not only offers disability pension to those who need it but also progressive rehabilitation programs or an adapted version of supported employment for patients who want to go back to work, is well adapted to some TIPP patients' needs. The disability pension has a very strong financial structure and has made a major effort towards early detection of unemployment linked to ill‐health and it is therefore likely to be complementary to RESSORT. Indeed, IPS's goal is to attain rapidly the primary job market, but some patients, even with a high level of functioning, do need longer periods of training before this aim is realistic.

## 5 Limitations

This study has many limitations. First, a high number of patients had to be excluded from the analysis, mainly due to lack of data on symptoms which was a domain we wanted to explore. However, we could check that none of these patients had contact with or entered the RESSORT program. Second, the sample of participants addressed to RESSORT was small, which limited the statistical power. This enhanced the risk of not detecting existing differences. Third, our assessment of functioning at the end of TIPP program was rather superficial and did not allow us to compare number of hours worked and type of job that patients found. The suggestion that RESSORT users had a better return to premorbid functional level and that the offer developed by DI had a similar impact that RESSORT on other functional outcomes needs validation based on a larger sample and a more detailed assessment. Fourth, the time elapsed between referral to RESSORT and the time of assessment was not available either. Although it may explain why patients had found another solution when assessed by RESSORT, a more detailed study would be needed to clarify this point. Finally, although the RESSORT team attempted to operationalise admission criteria, the referral of TIPP patients to this program depended importantly on the case manager's decision which was likely to be influenced by their difficulty guaranteeing a 50% working capacity and by the length of the waiting list for example. This context induces a systemic bias that we can't account for.

## 6 Conclusions

Despite these limitations, this study suggests that access to IPS for patients with a first episode of psychosis is majorly hampered if such programs are not specifically adapted to the specific needs and characteristics of patients in the early phases of psychosis. Integrating skills of supported employment within the role of case managers in the early psychosis program would have many advantages: fully integrating professional issues into care, better identifying and motivating candidates as part of follow‐up, offering direct access to the service without having to go through a third party with dissuasive inclusion criteria, and finally, better adapting the admission criteria and the mission of supported employment to the needs of the target population. To better adapt to the needs of people with early psychosis in a Swiss context, the supported employment program should offer: supported education and supported internships, the same financial and support benefits as disability insurance programs, no minimum activity rate requirement, support for undocumented workers engaged in undeclared work, and inclusion of individuals with a pending disability insurance claim. While other approaches, such as in our case disability insurance job coaching, may be complementary to IPS, the well‐established efficacy of IPS (Killackey et al. 2019) and the results of the present study suggest that an effort should be made to implement IPS programs within early intervention programs in order for this useful offer to be available to all FEP patients.

## Funding

The authors have nothing to report.

## Conflicts of Interest

The authors declare no conflicts of interest.

## Data Availability

The data that support the findings of this study are available on request from the corresponding author. The data are not publicly available due to privacy or ethical restrictions.
